# Activation of type I interferon antiviral response in human neural stem cells

**DOI:** 10.1186/s13287-019-1521-5

**Published:** 2019-12-16

**Authors:** Jhao-Yin Lin, Rei-Lin Kuo, Hsing-I Huang

**Affiliations:** 1 Research Center for Emerging Viral Infections, College of Medicine, Chang Gung University, Kwei-Shan, Tao-Yuan, Taiwan; 2 Graduate Institute of Biomedical Sciences, College of Medicine, Chang Gung University, Kwei-Shan, Tao-Yuan, Taiwan; 3 Department of Medical Biotechnology and Laboratory Science, College of Medicine, Chang Gung University, Kwei-Shan, Tao-Yuan, Taiwan; 4 Department of Pediatrics, Chang Gung Memorial Hospital, Linkou, Taiwan

**Keywords:** Neural stem cells, RIG-I, MDA5, IFN, Zika virus

## Abstract

**Background:**

Neural stem cells (NSCs) residing in the central nervous system play an important role in neurogenesis. Several viruses can infect these neural progenitors and cause severe neurological diseases. The innate immune responses against the neurotropic viruses in these tissue-specific stem cells remain unclear.

**Methods:**

Human NSCs were transfected with viral RNA mimics or infected with neurotropic virus for detecting the expression of antiviral interferons (IFNs) and downstream IFN-stimulated antiviral genes.

**Results:**

NSCs are able to produce interferon-β (IFN-β) (type I) and λ1 (type III) after transfection with poly(I:C) and that downstream IFN-stimulated antiviral genes, such as ISG56 and MxA, and the viral RNA sensors RIG-I, MDA5, and TLR3, can be expressed in NSCs under poly(I:C) or IFN-β stimulation. In addition, our results show that the pattern recognition receptors RIG-I and MDA5, as well as the endosomal pathogen recognition receptor TLR3, but not TLR7 and TLR8, are involved in the activation of IFN-β transcription in NSCs. Furthermore, NSCs infected with the neurotropic viruses, Zika and Japanese encephalitis viruses, are able to induce RIG-I-mediated IFN-β expression.

**Conclusion:**

Human NSCs have the ability to activate IFN signals against neurotropic viral pathogens.

## Background

Innate immune pathways play important roles in defending cells against pathogenic virus infections. These invading viruses are recognized by pattern recognition receptors (PRRs) to activate the expression of type I interferons (IFNs), which exert antiviral effects through upregulating the expression of IFN-stimulated genes (ISGs), such as ISG54 and 56 and MxA [1]. Therefore, type I IFNs have been used as therapeutic agents against viral infections in humans [2, 3]. In addition, type III IFNs (i.e., IFN-λs), which can be induced by PRR signaling to activate antiviral responses in cells, have also been used as antiviral agents to treat chronic viral infections [4, 5].

PRRs, such as cytoplasmic retinoic acid-inducible gene I (RIG-I)-like receptors and transmembrane Toll-like receptors (TLRs), recognize viral nucleic acids to initiate the IFN signaling pathway. RIG-I-like receptors comprise RIG-I and MDA5. RIG-I recognizes 5′-triphosphate (5′ppp) containing short double-stranded RNA, whereas MDA5 senses long double-stranded RNA or highly ordered RNA structure [6–8]. TLR3 also senses long double-stranded RNA, while TLR7 and TLR8 detect U-rich and GU-rich single-stranded RNA, respectively [9]. Because of these properties, PRRs vary in recognizing different RNA viruses. Previous studies reported that RIG-I is involved in detecting orthomyxoviruses, paramyxoviruses, and flaviviruses, whereas MDA5 is involved in the recognition of picornaviruses [10, 11]. However, both RIG-I and MDA5 are involved in the activation of the innate immune response during West Nile virus infection [12, 13], while the genome of dengue virus can only be recognized by RIG-I but not MDA5 [14]. In addition, the 5′ region of the Zika virus (ZIKV) genome is an RIG-I agonist [14]. The expression of PRRs could also be cell type- or tissue-specific [15, 16]. For example, TLR7 could be highly expressed in human plasmacytoid dendritic cells (pDCs) compared to other circulating immune cells and non-immune cells [17, 18]. The function and expression of RIG-I-like receptors and TLRs may also be species-specific [19, 20]. These studies suggest that the functions of PRRs may differ by cell types and species.

The central nervous system (CNS) has been thought to be an immune-privileged area. However, previous studies have shown that the cells residing in the CNS express PRRs [21]. For example, TLR3 and TLR8 are expressed by cortical and dorsal root ganglion neurons, while TLR7 is highly expressed in the spinal cord [15, 22]. In addition, the cytoplasmic PRRs RIG-I and MDA5 are expressed in human neural cells [23]. Microglia and astrocytes are able to produce RIG-I and MDA5, which may function as innate immune response mediators [24, 25]. Furthermore, several studies have shown that the expression of IFN-β is upregulated in the brains of mice infected with La Cross virus and Theiler’s murine encephalomyelitis virus [26, 27]. Therefore, PRR-mediated innate immune responses could be evoked in the CNS during viral infection.

The expression and function of PRRs vary in different stem cells. Several TLRs have been identified in mesenchymal stem cells (MSCs) to regulate proliferation and differentiation of the cells [28, 29]. In addition, the expression of RIG-I-like receptors (RLRs) RIG-I and MDA5 can be activated in MSCs after poly(I:C) transfection, and the induced expression of the RLRs stimulated IFN-β production and apoptosis in MSCs [30]. However, TLR3 and MDA5 are not expressed in embryonic stem cells (ESCs), though RIG-I is expressed but not functional in stimulating the innate immune response in ESCs [31]. Neural stem cells (NSCs), which are localized in the subventricular zone of the brain, are progenitors for all neural lineage cells. Previous studies demonstrated that TLR2 and TLR4 are expressed in adult NSCs and are involved in self-renewal of the cells [32] and that TNF-α can be secreted from these undifferentiated stem cells under stimulation by TLR2 and TLR4 agonists [33]. In addition, TLR3 is expressed in NSCs to negatively regulate the proliferation of the cells [34].

Although accumulating evidence shows that NSCs are susceptible to various neurotropic viruses, such as ZIKV, Japanese encephalitis virus (JEV), and herpes simplex virus [35–38], it remains unclear whether infections of these viruses can evoke innate immune responses. In this study, we examined IFN responses in human NSCs triggered by various agonists or infection with ZIKV or JEV. The results show that the transcription of type I and III IFNs was increased during poly(I:C) transfection. In addition, the expression of the IFN-stimulated antiviral genes, such as ISG56 and MxA, and the viral RNA sensors RIG-I, MDA5, and TLR3 are induced in NSCs by poly(I:C) transfection or IFN-β treatment. We further reveal that RIG-I, MDA5, and TLR3 function in response to their specific ligands for stimulating IFN-β transcription in NSCs. Moreover, our results demonstrate that infection of ZIKV with NSCs dramatically induces the expression of IFN-β in the RIG-I-dependent pathway. This work provides insights into the innate immune response in human NSCs during neurotropic virus infection.

## Methods

### Cells and virus

Human NSCs derived from the NIH-approved H9 hESCs were obtained from Invitrogen (Thermo-Fisher Scientific, MA, USA). Cells were cultured in a complete medium consisting of 1× KnockOut™ D-MEM/F12, 2 mM GlutaMAX™-I Supplement, 20 ng/ml b-FGF, 20 ng/ml EGF, and 2% StemPro^(R)^ neural supplement (all from Thermo-Fisher Scientific, MA, USA). Culture dishes were coated with CELLStart™ (Thermo-Fisher Scientific, MA, USA) before use. The cells were incubated at 37 °C and 5% CO_2_ in a humidified incubator. THP-1 cell (BCRC number 60430) was purchased from the Bioresource Collection and Research Center (BCRC), Taiwan. THP-1 cells were cultivated in RPMI medium supplemented with 10% FBS. Vero cell was provided by Dr. Shin-Ru Shih at Chang Gung University. Vero cells were cultivated in DMEM medium supplemented with 10% FBS, 1% non-essential amino acid, 1% l-glutamine, and 1% penicillin/streptomycin (all from Thermo-Fisher Scientific, MA, USA). Human peripheral blood cells were collected from healthy volunteers with informed consent approved by an institutional review board (Chang Gung Medical Foundation institutional review board, IRB: 201800368B0). ZIKV (PRVABC59) was obtained from the Center for Disease Control, Taiwan. JEV (T1P1) was a kind gift obtained from Prof. Robert, Y.-L. Wang at Chang Gung University.

### Virus infection

For virus infection, cells were seeded in culture plates for approximately 24 h before infection. After washing, the virus was added to the cells at a specified multiplicity of infection (MOI). After 2 h of adsorption, unbound viral particles were removed, and fresh medium was added for subsequent incubation.

### Preparation of reagents

Poly(I:C) (Sigma-Aldrich, MO, USA) was prepared at 5 mg/ml in PBS. Poly(I:C) HMW/LyoVecTM (InvivoGen, CA, USA) was prepared at 0.125 mg/ml in endotoxin-free water. Poly(A:U) (InvivoGen, CA, USA) was prepared at 1 mg/ml in sterile physiologic water. Motolimod and GS9620 (Selleckchem, MA, USA) were prepared at 1 mM in DMSO. IFN-β recombinant protein (Peprotech, NJ, USA) was prepared at 1 mg/ml in sterilized water.

### Preparation of 5′pppRNA

To synthesize 5′pppRNA, the linear pcDNA3 plasmid digested with NotI was used for in vitro transcription. The MEGAscript T7 Kit (Thermo-Fisher Scientific, MA, USA) was used to synthesize the RNA transcripts. One microgram of linearized DNA templates was mixed with 75 mM ATP, 75 mM UTP, 75 mM CTP, 75 mM GTP (2 μl of each nucleotide), 2 μl of 10× reaction buffer, 2 μl of enzyme mix, and nuclease-free water to a volume of 20 μl. After incubation at 37 °C for 4 h, TURBO DNase (2 U/l) was added for another 15 min. Synthesized RNAs were then purified with RNeasy Protect Minikit (Qiagen, Hilden, Germany) and stored at − 80 °C.

### Cell transfection

Cells were seeded in CELLStart-coated plates and incubated in medium until 90% confluence. Lipofectamine 2000 (Thermo-Fisher Scientific, MA, USA) and reagents A and B were prepared according to the manufacturer’s protocol. Reagent A was prepared by mixing 100 μl of opti-MEM with 1 μg of poly(I:C), 1 μg of 5′pppRNA, 1 μg of poly(I:C) HMW, 1 μg of poly(A:U), GS9620 (1 μM), and motolimod (20 μM). Two microliters of Lipofectamine 2000 was diluted in 100 μl opti-MEM for reagent B preparation. Reagents A and B were mixed and incubated at room temperature for 20 min. The mixtures were then added into tested cells for transfection.

### Knockdown assay

Specific siRNAs (Sigma-Aldrich, MO, USA) were prepared with a concentration of 100 μM using RNase-free distilled water. Lipofectamine 2000 RNAiMAX (Thermo-Fisher Scientific, MA, USA) was used for transfecting siRNAs. Reagent A containing siRNA diluted in 100 μl opti-MEM and reagent B containing 4 μl Lipofectamine 2000 RNAiMAX diluted in 100 μl opti-MEM were prepared. Reagents A and B were mixed and incubated at room temperature for 5 min. The prepared mixtures were then added to the cells and incubated at 37 °C, 5% CO_2_ for 6 h. The old medium was decanted, and fresh expansion medium was added for incubation. The knockdown efficiencies were examined after 72 h of transfection by immunoblot analysis.

### IFN-β ELISA assay

The supernatant of human neural stem cells transfected with poly(I:C) was harvested at 6, 12, and 24 h at post-transfection. The supernatant was centrifuged at 5000 rpm, 10 min, 4 °C for removing cell debris. The expression of IFN-β protein was detected with VerKine™ human IFN-beta ELISA kit (PBL Assay Science, NJ, USA). According to the manufacturer’s instruction, the 50-μl sample diluent, standard and samples were added to the wells and incubated at RT for 1 h. The contents of wells were aspirated, and the wells were washed three times with wash buffer. The 100-μl diluted antibody solution was added to the wells and incubated at RT for 1 h, to aspirate the antibody solution and wash the wells. The 100-μl diluted HRP solution was added to the wells and incubated at RT for 1 h, to aspirate the HRP solution and wash the wells. The 100-μl TMB substrate solution was added to the wells and incubated at RT for 15 min and then 100-μl stop solution was added to wells to stop the reaction and to read the plated at 450 nm absorbance by Synergy 2 Multi-Mode microplate reader (BioTek, VT, USA).

### IFN-β antibody blocking assay

Human neural stem cells were seeded in 24-well plates coated by CELLStart and grew to about 90% confluent in complete medium. The cells were infected with ZIKV at an MOI of 1, and then the IFN-β antibody (Thermo-Fisher Scientific, MA, USA) was added in the new complete medium after virus adsorption. The total RNA was harvested, and RT-qPCR was performed to detect viral replication at 24 h post-infection.

### RNA isolation and RT-qPCR

Total RNA was isolated using TRIzol reagent (Life Technologies, CA, USA). One microgram of total RNA was used to synthesize cDNA using the RevertAid First Strand cDNA Synthesis Kit (Thermo-Fisher Scientific, MA, USA) according to the manufacturer’s instructions. DNase I (Promega, WI, USA) was used to remove genomic DNA, and 50 mM EDTA was used to inactivate DNase I. A total of 1 μl of cDNA sample with 5 μM primers and SYBR green (KAPA Biosystems, MA, USA) was used to perform qPCR. qPCR assays were carried out in 384-well plates and analyzed by the Roche Light Cycler 480 (Roche, Basel, SW). Each sample was assayed in triplicate, and 18 s rRNA was used as a reference gene. The relative quantification of each gene was calculated by the 2^−ΔΔCT^ method. The primers used in the assay are shown in Additional file 5: Table S1.

### Plaque assay

Vero cells were expanded in DMEM containing10% FBS and seeded on 6-well plate at the concentration of 5 × 10^5^ cells/well. After incubation overnight, the cells were infected by serially diluted virus solution. After 2 h of adsorption, the virus suspension was removed and washed twice with PBS. DMEM supplemented with 2% FBS and 0.3% agarose was then added to the cells. Cells were fixed with 10% formaldehyde for 1 h after 5 days of incubation. The agarose medium was rinsed out, and the fixed cells were stained with 0.5% crystal violate.

### Western blot

The cultured cells were washed with PBS and lysed with ice-cold protein lysis buffer (1% NP-40, 50 mM Tris, and 150 mM NaCl) supplemented with 1× protease inhibitor cocktail. The cell lysate was incubated on ice for 30 min and centrifuged at 13,000 rpm for 10 min at 4 °C. The Bradford method (Bio-Rad Laboratories, CA, USA) was used to measure the protein concentration of the supernatant. Protein samples were separated by 8 or 12% SDS-polyacrylamide gel electrophoresis and then transferred onto a polyvinylidene fluoride membrane (PVDF) (GE, MA, USA). The protein-containing membrane was blocked with 5% skim milk in Tris-buffered saline Tween-20 (TBST, 20 mmol/ml Tris-HCl, pH 7.4, 150 mmol/l NaCl, and 0.1% Tween-20) at room temperature. The membrane was then incubated with rabbit anti-RIG-I (1:1000, Pro-Sci, CA, USA), rabbit anti-MDA5 (1:2000, Enzo, NY, USA), rabbit anti-TLR3 (1:1000, Abcam, Cambridge, UK), rabbit anti-IRF3 (1:1000, Santa Cruz, CA, USA), rabbit anti-phosphorylated IRF3-Ser396 (1:1000, Cell signaling, CA, USA), rabbit anti-phosphorylated IRF7-Ser471/472 (1:1000, Cell signaling, CA, USA), or mouse anti-β-actin (1: 20000, Sigma-Aldrich, MO, USA). Subsequently, the membrane was probed with anti-mouse or anti-rabbit secondary antibody conjugated with horseradish peroxidase (1:5000, Jackson ImmunoResearch Laboratories, PA, USA). The protein was detected with a chemiluminescence reagent (PerkinElmer, MA, USA) and a Chemi™ imaging system (Bio-Rad, CA, USA).

### Immunofluorescence assay

Seeded NSCs were washed with PBS and fixed with ice-cold 4% paraformaldehyde for 15 min at room temperature. The cells were then permeabilized with 0.5% Triton X-100 in TBS for 5 min and blocked with 2% FBS in TBS for 30 min at room temperature. The cells were incubated with primary antibodies: rabbit anti-sox2 (1:200, cell signaling, CA, USA), mouse anti-nestin (1:200, Millipore, MA, USA), rabbit anti-MAP2 (1:200, Millipore, MA, USA), mouse anti-β-tubulin III (1:200, Millipore, MA, USA), rabbit anti-GFAP (1:200, Stem Cell Technologies, BC, CA), rabbit anti-phosphorylated IRF3 (Ser396) (1:200, cell signaling, CA, USA), or mouse anti-ZIKV E protein (1:500, GeneTex, CA, USA) at 4 °C overnight. The cells were then washed three times with TBS and incubated with Dylight 594-conjugated donkey anti-mouse secondary antibody or Dylight 488-conjugated goat anti-rabbit secondary antibody (1:1000, Jackson ImmunoResearch Laboratories, PA, USA) for 1 h at room temperature. The cells were then washed three times with TBS, and the cell nuclei were counterstained with DAPI (4′,6-diamidino-2-phenylindole) (Sigma-Aldrich, MO, USA). The images were collected with a fluorescence microscope (Olympus BX51, Olympus, Tokyo, JP).

### Cellular viability assay

MTT [3-(4,5-dimethylthiazol-2-yl)-2,5-diphenyltetrazolium bromide] (Sigma-Aldrich, MO, USA) was prepared in PBS to get a stock solution (5 mg/ml). The culture medium was removed, and MTT solution diluted in complete medium (1 mg/ml) was added to the cells. The cells were then incubated at 37 °C, 5% CO_2_ for 4 h. The MTT solution was removed, and the violet formazan crystals were dissolved with 0.04 N HCl in isopropanol. The absorbance was measured at 570 nm with an ELISA reader (BioTek, VT, USA).

### Statistical analysis

Measurement data were expressed as the mean ± standard deviation (SD). Statistical significance was determined by Student’s *t* test or two-way ANOVA.

## Results

### Characterization of human NSCs

**Fig. 1:**
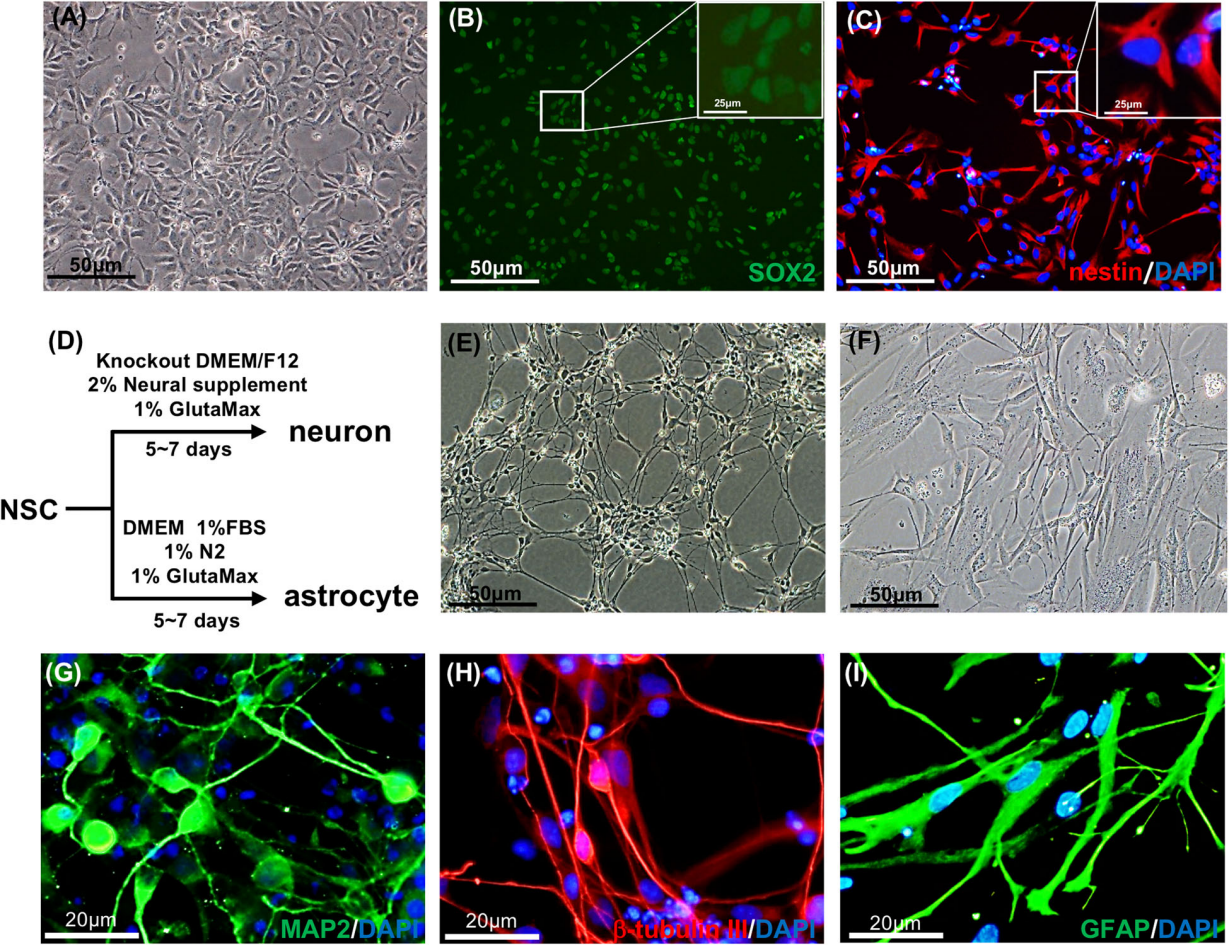
Characterization of human neural stem cells. **a** Bright-field image of hNSCs. Scale bar = 50 μm. Immunostaining images of hNSCs showing the expression of SOX2 (**b**) and nestin (**c**). The nuclei are counterstained by DAPI. Scale bar = 50 μm. **d** Schematic diagram of neuronal and astrocytic differentiation from hNSCs. Bright-field images of neurons (**e**) and astrocytes (**f**) derived from hNSCs. Scale bar = 50 μm. Immunostaining images of hNSC-differentiated neuronal cells showing the expression of MAP2 (**g**) and β-tubulin III (**h**). The nuclei were counterstained by DAPI. Scale bar = 20 μm. **i** Immunofluorescence results in hNSC-converted astrocytic cells showing the expression of GFAP. The nuclei were counterstained by DAPI. Scale bar = 20 μm

To characterize human NSCs, the cells were expanded (Fig. 1a), and the expression of SOX2 and nestin was analyzed by immunofluorescence staining. The results showed that all cells expressed these two markers (Fig. 1b, c). In addition, the neuronal and glial differentiation potentials were analyzed by in vitro differentiation procedures (Fig. 1d). After treatment with the differentiation media, the cell morphology changed drastically. The differentiated neuron-like cells showed concentrated cell bodies with elongated dendrites, while astrocyte-like cells expressed branched processes (Fig. 1e, f). Immunofluorescent staining was used to confirm the differentiation. The differentiated neuronal cells positively expressed MAP2 and β-tubulin III, which are usually used to identify the postmitotic neurons (Fig. 1g, h). Furthermore, GFAP was detected in differentiated astrocyte-like cells (Fig. 1i). The results demonstrated the characteristics and differentiation potentials of the human NSCs used in this research.

### Activation of type I and III IFN expression in human NSCs

**Fig. 2:**
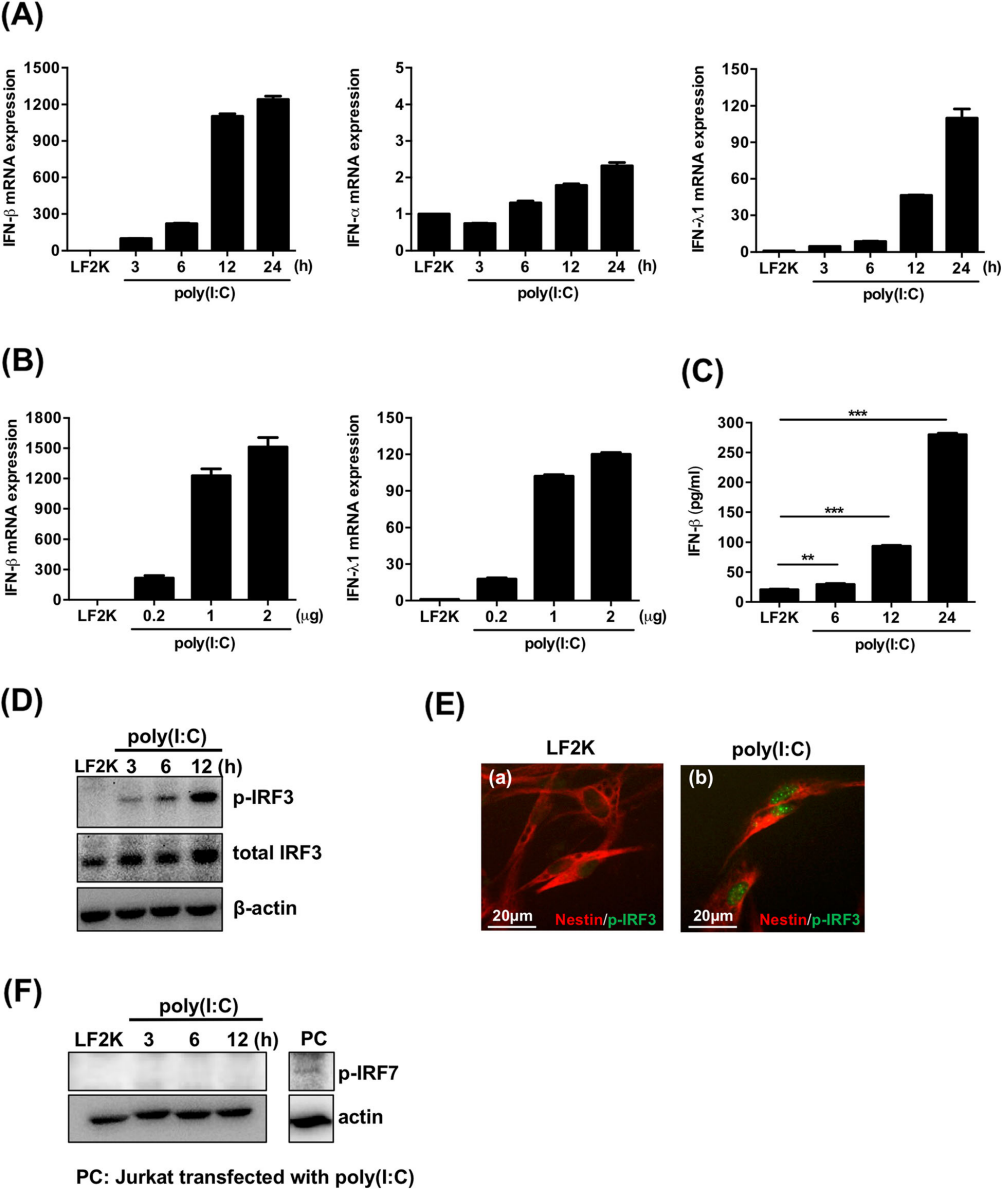
Poly(I:C) transfection upregulates mRNA expression of IFN-β and λ1 in hNSCs. **a** RT-qPCR analysis of IFN-β, α, and λ1 expression in poly(I:C)-transfected hNSCs (1 μg). The experiments were triplicated, and the error bars represented the standard deviation (SD). **b** Human NSCs were transfected with 0.2, 1, and 2 μg of poly(I:C) by Lipofectamine 2000 (LF2K), and the expression of IFN-β and IFN-λ1 was analyzed by RT-qPCR. The experiments were performed in triplicate, and the error bars represented the SD. **c** The supernatants of human NSCs transfected with 1 μg poly(I:C) were harvested at 6, 12, and 24 h post-transfection, and the protein expression of IFN-β was determined using ELISA. The experiments were performed in triplicate, and the error bars represented the SD. Student’s *t* test was used for statistical analysis. *p < 0.05, **p < 0.01, ***p < 0.001. **d** Western blot analysis was applied to examine the expression of phospho-IRF3 (p-IRF3) and total IRF3 at different time points in poly(I:C)-transfected hNSCs (1 μg). **e** Immunofluorescence images of p-IRF3 and nestin in human NSCs transfected with LF2K only (**a**) and poly(I:C) (**b**). The cell nuclei were counterstained with DAPI. Scale bar = 20 μm. **f** Western blot analysis was applied to examine the expression of phospho-IRF7 (p-IRF7) at different time points in poly(I:C)-transfected hNSCs (1 μg). β-actin was used as an internal control

To investigate the innate immunity of human NSCs in response to viral infections, the capability of IFN induction of the cells was examined under specific agonist stimulation. Initially, the human NSCs were transfected with poly(I:C) for 3, 6, 12, and 24 h, and then the mRNA expression of IFN-α/β (type I) and λ1 (type III) was detected. We found that mRNA levels of IFN-β and λ1 significantly increased in human NSCs transfected with poly(I:C), whereas IFN-α was not significantly increased (Fig. 2a). In addition, the increased transcriptional activation of IFN-β and λ1 stimulated by poly(I:C) in human NSCs was confirmed by transfection of poly(I:C) in a dose-dependent manner (Fig. 2b). Consistent with the mRNA expression, the protein levels of IFN-β were significantly increased in human NSCs transfected with poly(I:C) (Fig. 2c). Since the activation of type I IFN transcription is dependent on the activation of IRF3, we further determined IRF3 activation by examining the phosphorylated form (Ser-396) of IRF3 in the transfected cells. As shown in Fig. 2d, phosphorylated IRF3 was detected in the extracts of NSCs transfected with poly(I:C). We consistently observed phosphorylated IRF3 in the poly(I:C)-transfected NSCs by immunofluorescence (Fig. 2e). Although activation of IRF7 could also upregulate the activation of type I IFN, we did not detect the phosphorylated IRF7 in human NSCs transfected with poly(I:C) (Fig. 2f). The results demonstrate that human NSCs are competent to express type I and III IFNs in response to stimulation of dsRNA analogs.

### Human NSCs are competent in the activation of antiviral gene transcription

**Fig. 3:**
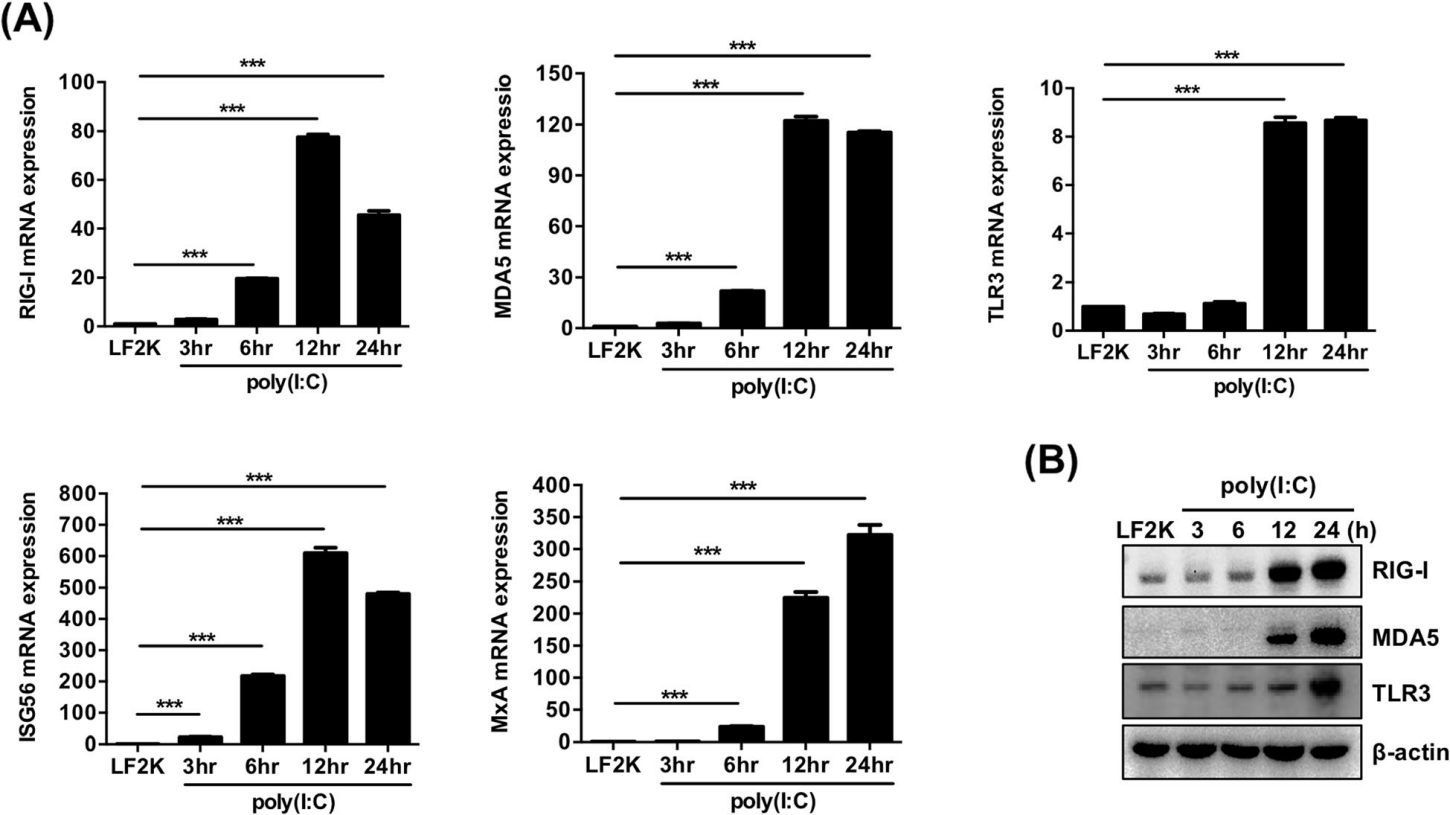
Expression of RIG-I, MDA5, TLR3, ISG56, and MxA is upregulated in poly(I:C)-transfected hNSCs. **a** RT-qPCR analysis was performed to detect the expression of RIG-I, MDA5, TLR3, ISG56, and MxA in human NSCs transfected with 1 μg poly(I:C) for the indicated time. The experiments were performed in triplicate, and the error bars represented the SD. Student’s *t* test was used for statistical analysis. *p < 0.05, **p < 0.01, ***p < 0.001. **b** Immunoblot analysis was performed to detect the expression of RIG-I, MDA5, and TLR3 in poly(I:C)-transfected human NSCs. β-actin was used as an internal control

Our previous results have demonstrated that type I IFNs can be induced in human NSCs. We further determined whether the downstream antiviral genes activated by type I IFN can be expressed in NSCs by detecting mRNA of the ISGs, including ISG56 and MxA, and viral RNA sensors RIG-I, MDA5, and TLR3. As shown in Fig. 3a, mRNA expression of the ISGs increased in the poly(I:C)-transfected NSCs. The immunoblots also probed the increasing protein expression levels of RIG-I, MDA5, and TLR3 (Fig. 3b). In addition, the NSCs were treated with IFN-β for 1, 2, 3, and 4 h, and then the mRNA expression of RIG-I, MDA5, TLR3, ISG56, and MxA was detected. As shown in Additional file 1: Figure S1A, treatment with IFN-β dramatically increased the mRNA levels of the aforementioned antiviral ISGs. Increasing amounts of RIG-I, MDA5, and TLR3 were also observed in the IFN-β-treated NSCs (Additional file 1: Figure S1B). The effects were further confirmed by treating NSCs with two different doses of IFN-β (Additional file 1: Figure S1C and D). Hence, the results imply that the activation of type I IFN in NSCs results in the induction of downstream antiviral gene expression.

### The role of pathogen recognition receptors in human NSCs in the production of type I IFN

**Fig. 4:**
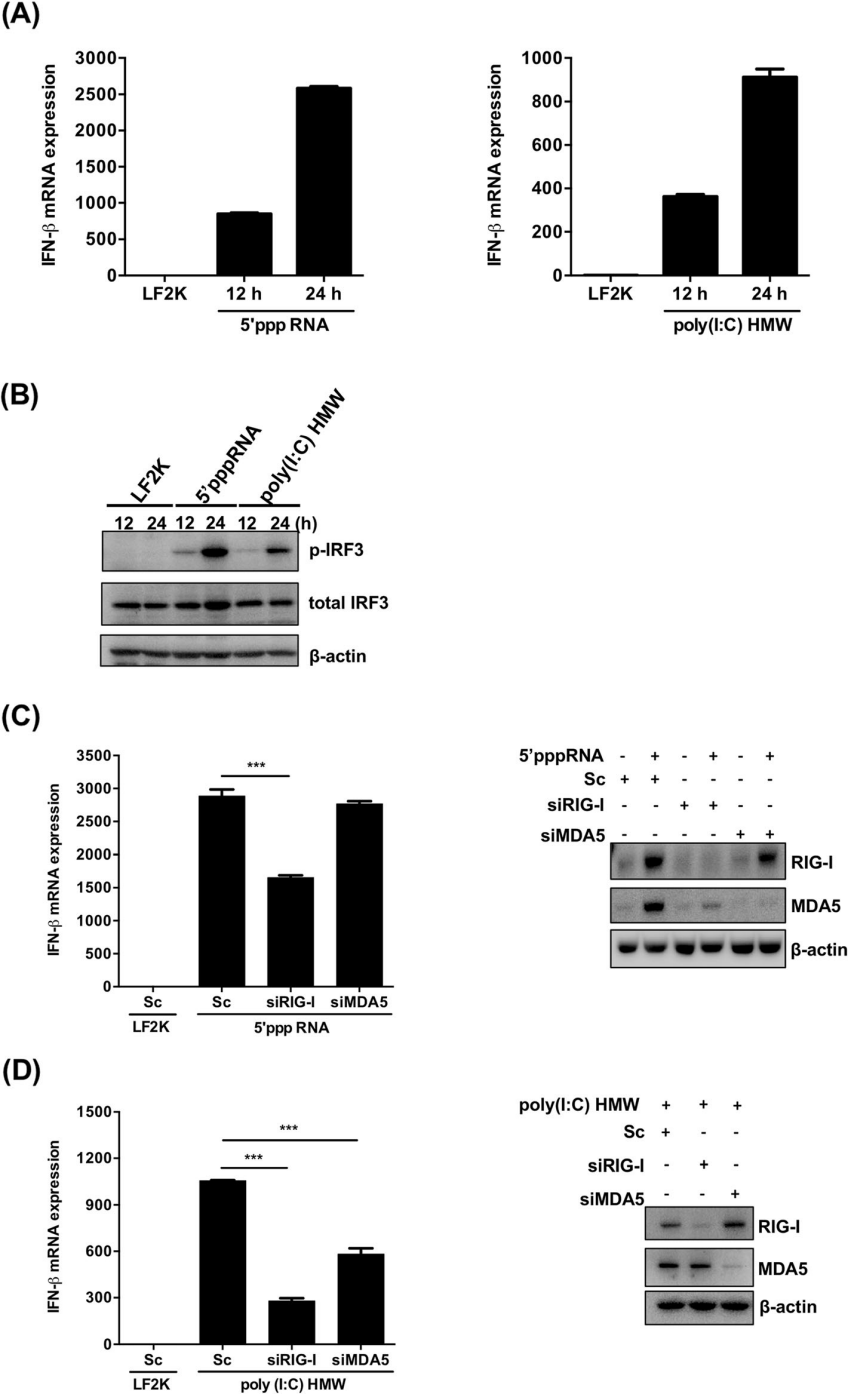
RIG-I and MDA5 agonists stimulate the expression of IFN-β in hNSCs. **a**, **b** Human NSCs were transfected with 5′pppRNA or poly(I:C) HMW. Total RNA was collected at 12 and 24 h post-transfection, and RT-qPCR assay was performed to examine the relative amount of IFN-β mRNA (**a**). The expression of phospho-IRF3 and total IRF3 was detected through a western blot assay. β-actin was used as an internal control. (**b**). **c** Human NSCs were transfected with siRNA specific for RIG-I or MDA5 by Lipofectamine RNAiMAX 2000 and then transfected with 1 μg 5′pppRNA for 24 h. RT-qPCR analysis was performed to detect the expression of IFN-β transcripts while western blot was applied to confirm the knockdown efficiency. **d** Human NSCs were transfected with siRNA targeting RIG-I or MDA5 and then transfected with 1 μg of poly(I:C) HMW for 24 h. RT-qPCR analysis was performed to detect the expression of IFN-β transcripts while western blot was applied to confirm the knockdown efficiency. The experiments were performed in triplicate, and the error bars represented the SD. Student’s *t* test was used for statistical analysis. *p < 0.05, **p < 0.01, ***p < 0.001

Previous results have shown that the expression of viral RNA sensors, including cytoplasmic PRRs RIG-I and MDA5 and the endosomal viral RNA sensor TLR3, could be induced in human NSCs by transfection of poly(I:C) (Fig. 3). These results indicate that these pathogen sensors can be normally expressed and induced by the pathogen-associated molecular pattern (PAMP) in human NSCs. To further determine the role of the PRRs in activating type I IFN signaling, specific ligands of the PRRs were used to stimulate the transcription of IFN-β in human NSCs. The NSCs were individually transfected with in vitro-transcribed RNA with 5′-triphosphate (5′pppRNA) and poly(I:C) high molecular weight (HMW), which are the ligands of RIG-I and MDA5, respectively. At 12 and 24 h post-transfection, total RNA of the transfected NSCs was collected and subjected to RT-qPCR to detect IFN-β mRNA. We found that the expression of IFN-β mRNA in NSCs was enhanced by the transfection of the ligands (Fig. 4a). In addition to measuring IFN-β mRNA, activation of IRF3 in the NSCs stimulated by the specific ligands was determined by immunoblotting of phosphorylated IRF3. The results demonstrated that IRF3 was consistently activated by RIG-I and MDA5 ligands, 5′pppRNA and poly(I:C) HMW, respectively, which dramatically induced IFN-β transcription (Fig. 4b).

To validate the role of RIG-I and MDA5 in activating the innate immune response in NSCs, we examined IFN-β transcription under stimulation with specific agonists in NSCs with knocked down RIG-I or MDA5 expression. As anticipated, knockdown of RIG-I expression reduced the 5′pppRNA-activated transcription of IFN-β in the NSCs, while knockdown of MDA5 expression did not (Fig. 4c). Surprisingly, we found that the expression of IFN-β mRNA initiated by poly(I:C) HMW was decreased by knockdown of either RIG-I or MDA5 (Fig. 4d), suggesting that the long dsRNA ligand may be sensed by RIG-I or MDA5 in NSCs. Additionally, total RNA extracted from Enterovirus A71 (EV-A71)-infected cells, which was considered as a MDA5 ligand [39], was used to validate the result above. We found that IFN-β mRNA expression initiated by total RNA of EV-A71-infected cells was also decreased in the NSCs that were knocked down with either RIG-I or MDA5 siRNA (Additional file 2: Figure S2A), in contrast to the result obtained from HeLa cells (Additional file 2: Figure S2B). Collectively, the results demonstrate that the cytoplasmic PRRs RIG-I and MDA5 can be induced and serve the role for sensing their ligands in NSCs.

**Fig. 5:**
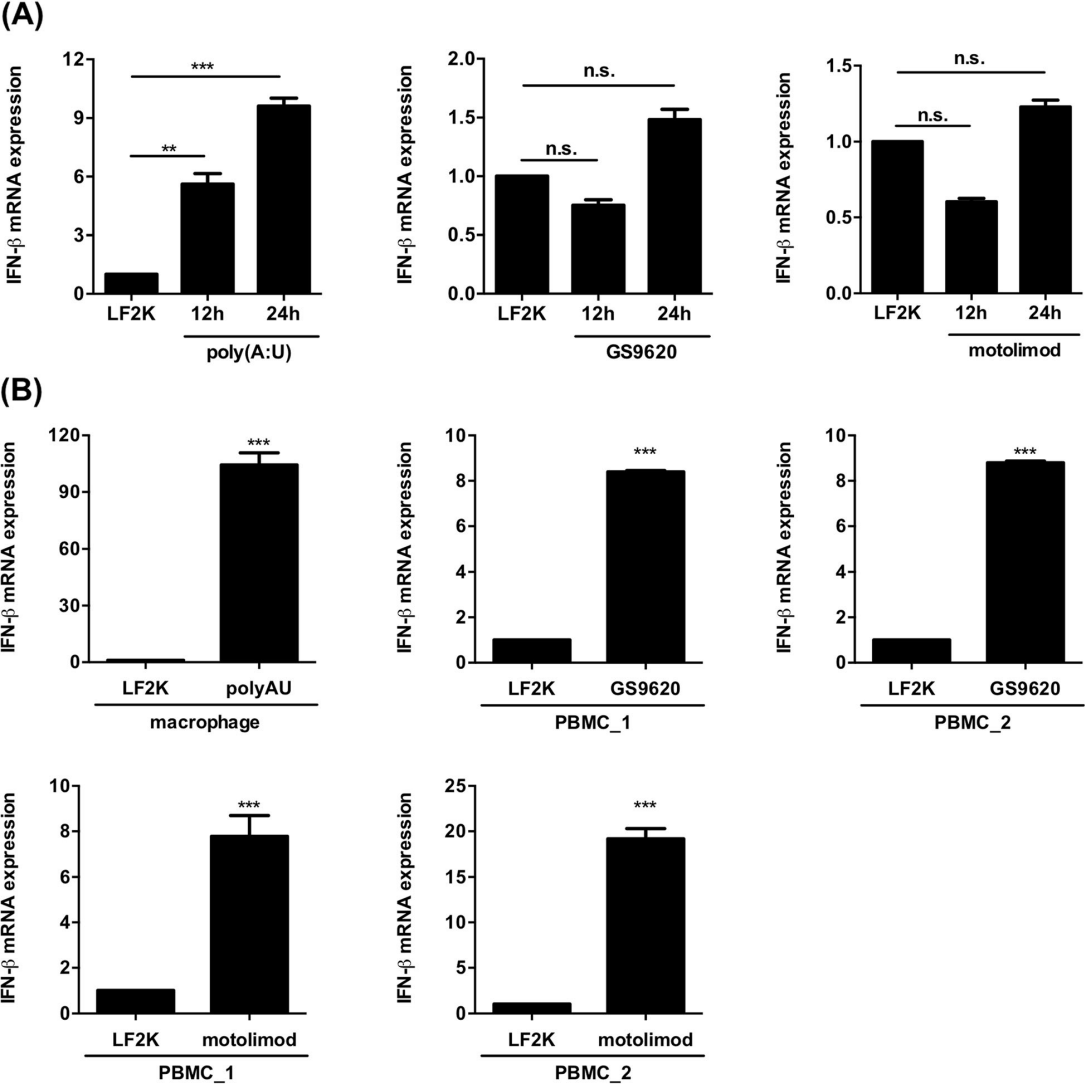
Poly(A:U), but not GS9620 and motolimod, is able to induce the IFN-β upregulation in hNSCs. **a** RT-qPCR analysis was performed to detect the IFN-β expression in NSCs transfected with poly(A:U) (1 μg), GS9620(1 μM), and motolimod (20 μM) for the indicated time points. **b** RT-qPCR analysis was performed to detect the IFN-β expression in PMA-primed THP-1 cells transfected with poly(A:U) (1 μg) and human PBMCs transfected with GS9620 (1 μM) and motolimod (20 μM). The experiments were performed in triplicate, and the error bars represented the SD. Student’s *t* test was used for statistical analysis. *p < 0.05, **p < 0.01, ***p < 0.001. n.s., non-significant

In addition, we examined the expression and function of the endosomal PRRs TLR3, TLR7, and TLR8 in NSCs. The NSCs were separately transfected with poly(A:U), GS9620, and motolimod, which are agonists of TLR3, TLR7, and TLR8, respectively. At 12 and 24 h after transfection, total RNA of the NSCs was collected to examine the expression of IFN-β mRNA. The results showed that stimulation with poly(A:U) could induce IFN-β mRNA expression in human NSCs. In contrast, the ligands of TRL7 and TLR8 only weakly induced transcription of IFN-β mRNA (Fig. 5a), though the ligands were potent enough to activate IFN-β mRNA expression in THP-1-derived macrophages and peripheral blood mononuclear cells (PBMC) (Fig. 5b). The results indicate that endosomal PRR TLR3, but not TLR7 or TLR8, is functional in the activation of IFN-β mRNA production in NSCs.

### Infection of ZIKV and JEV induces IFN-β expression mediated by RIG-I-like receptors in human NSCs

**Fig. 6:**
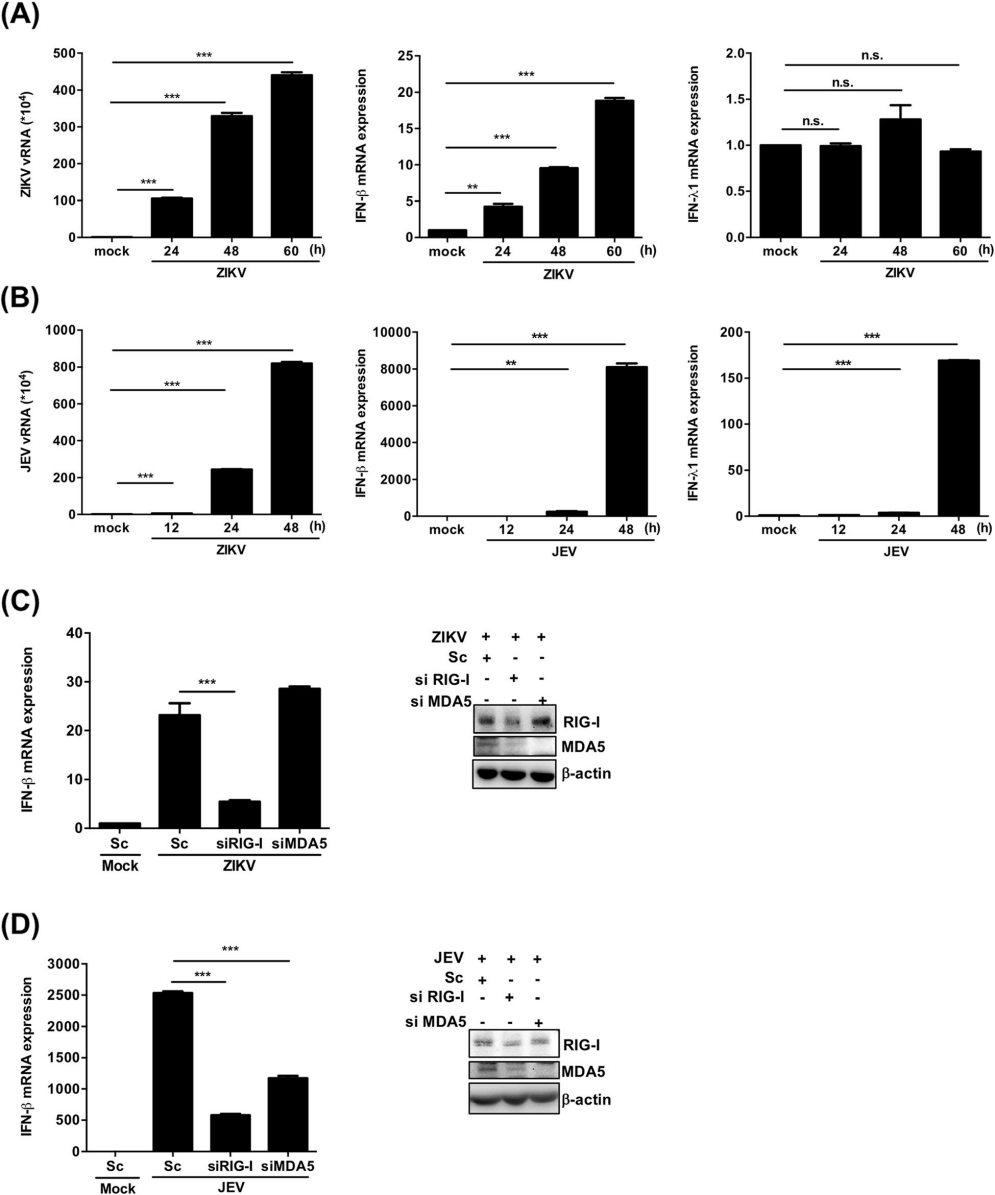
Transcription of IFN-β expression is increased in ZIKV- and JEV-infected hNSCs. **a** Human NSCs were infected with Zika virus at an MOI of 1, and RT-qPCR was applied to detect the mRNA levels of Zika virus RNA (vRNA), IFN-β, and IFN-λ1 at indicated time points. **b** RT-qPCR analysis was performed to examine the mRNA expression levels of JEV virus RNA, IFN-β, and IFN-λ1 in hNSCs that infected by JEV at an MOI of 1. **c** Human NSCs were transfected with siRNA targeting RIG-I or MDA5 and then infected with Zika virus at an MOI of 1 for 48 h. RT-qPCR was used to analyze the relative amounts of IFN-β mRNA, and western blot assay was performed to confirm the knockdown efficiency. **d** Human NSCs transfected with siRNA targeting RIG-I or MDA5 were infected with JEV at an MOI of 1 for 48 h. RT-qPCR analysis was performed to analyze the expression of IFN-β mRNA, and western blot assay was used to confirm the knockdown efficiency. The experiments were performed in triplicate, and the error bars represented the SD. Student’s *t* test was used for statistical analysis. *p < 0.05, **p < 0.01, ***p < 0.001. n.s., non-significant

ZIKV and JEV infect human CNS and cause severe neurological complications. It is critical to explore whether these neurological tropism viruses could infect human NSCs and elicit innate immune responses. Therefore, we examined the expression of type I and III IFNs in response to infection with these viruses. We found that both ZIKV and JEV could infect and replicate in NSCs, and the infection strongly induced IFN-β expression. IFN-λ1 expression was also strongly induced in JEV-infected NSCs, but not in ZIKV infection (Fig. 6a, b). In addition, knockdown of RIG-I, but not MDA5, decreased ZIKV-induced IFN-β mRNA expression in the NSCs, while JEV-induced IFN-β mRNA expression could be affected by knockdown of either RIG-I or MDA5 (Fig. 6c, d). The results demonstrate that ZIKV or JEV can be replicated and elicit the innate immune responses of type I IFN in human NSCs. Moreover, we conclude that RIG-I is involved in the activation of the type I IFN response for both ZIKV and JEV in NSCs. Interestingly, the result indicates that MDA5 may also play a role in JEV-induced IFN-β production in NSCs. We further examined the role of RIG-I and MDA5 in ZIKV replication in human NSCs. We demonstrated that the ZIKV replication was significantly increased in cells knocked down with RIG-I, but not MDA5 siRNA (Additional file 3: Figure S3). These results imply that RIG-I plays an important role in regulating the ZIKV replication through the induction of IFN-β in human NSCs.

### Activation of IFN-β signaling suppresses replication of Zika virus in human NSCs

**Fig. 7:**
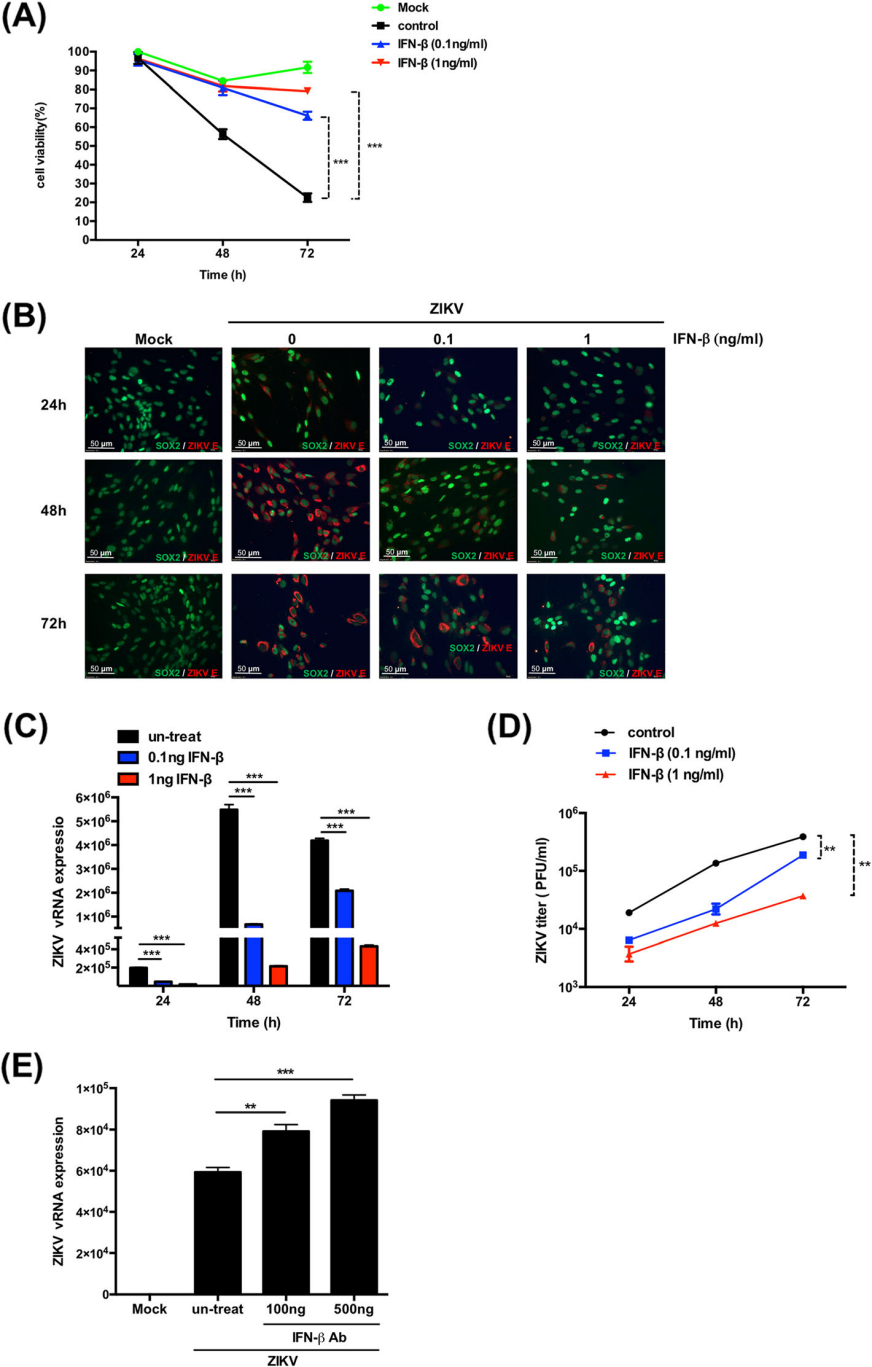
IFN-β treatment inhibits the replication of ZIKV in hNSCs. **a** Human NSCs were treated with different doses of IFN-β protein and then infected with ZIKV. The cellular viability was assessed by MTT assay. The experiments were performed in triplicate, and the error bars represented the SD. The two-way ANOVA was used for statistical analysis. *p < 0.05, **p < 0.01, ***p < 0.001. **b** Immunofluorescence staining was performed to detect the expression of ZIKV E protein in ZIKV-infected hNSCs that were treated with different doses of IFN-β protein. **c** RT-qPCR analysis was performed to detect ZIKV vRNA in ZIKV-infected hNSCs that treated with different doses of IFN-β. The experiments were performed in triplicate, and the error bars represented the SD. Student’s *t* test was used for statistical analysis. *p < 0.05, **p < 0.01, ***p < 0.001. **d** Growth curves of ZIKV in hNSCs treated with 0, 0.1, and 1 ng/ml IFN-β. The experiments were performed in triplicate, and the error bars represented the SD. The two-way ANOVA was used for statistical analysis. *p < 0.05, **p < 0.01, ***p < 0.001. **e** Human NSCs were infected with ZIKV at an MOI of 1, and then anti-IFN-β antibody (100 ng/ml and 500 ng/ml) was added to the cells after virus adsorption. Total RNA was harvested at 24 h post-infection, and RT-qPCR was performed to detect the virus RNA. The experiments were performed in triplicate, and the error bars represented the SD. Student’s *t* test was used for statistical analysis. *p < 0.05, **p < 0.01, ***p < 0.001

As shown previously, ZIKV infection induced RIG-I-mediated IFN-β transcription. We further determined whether the activation of IFN-β production could inhibit ZIKV replication. The NSCs were transfected with 5′pppRNA, which is an agonist for stimulating the RIG-I pathway, and then infected with ZIKV. We found that the NSCs transfected with the RIG-I agonist induced IFN-β transcription (Additional file 4: Figure S4A) and consequently inhibited the replication of ZIKV at 24 and 48 h after infection (Additional file 4: Figure S4B and C). In addition, the NSCs treated with IFN-β protein prior to ZIKV infection could maintain the viability from cell death caused by the infection (Fig. 7a). Correspondingly, IFN-β treatment suppressed the synthesis of ZIKV protein and RNA in the NSCs (Fig. 7b, c, respectively). We further confirmed the inhibitory effect by examining the production of infectious ZIKV in the supernatant of NSCs treated with IFN-β by plaque formation assay (Fig. 7d). For examining the effect of IFN-β secreted from ZIKV-infected human NSCs in viral replication, we treated the ZIKV-infected human NSCs with anti-IFN-β antibody to block the secreted IFN-β. IFN-β neutralization could increase the expression levels of ZIKV vRNA, compared to the untreated control (Fig. 7e). Collectively, the results demonstrate that activation of IFN-β transcription or direct treatment with IFN-β inhibits ZIKV replication in human NSCs. Based on the results, we conclude that human NSCs have an intact innate immune response in activating the RIG-I signaling pathway to counteract ZIKV infection.

## Discussion

NSCs are progenitors residing in specific areas that possess self-renewal and neuro-regenerating abilities. Recent studies have shown that NSCs are susceptible to infection by various neurotropic viruses, including ZIKV, JEV, EV-A71, and CVB3, which cause various deleterious neurological disorders [40–43]. However, little is known regarding whether the innate immune responses could be induced by viral pathogens in these cells and how the cells respond to IFNs. In the present study, we demonstrate that NSCs are able to produce IFN-β (type I) and λ1 (type III) upon transfection with poly(I:C), which is a synthetic analog of double-stranded RNA for mimicking viral infection. We also detected the activation of IRF3 in the poly(I:C)-transfected NSCs. However, IFN-α mRNA was not significantly induced in the human NSCs. It has been reported that activation of IRF7 plays an essential role for the transcription of IFN-α [44]. Consistently, we did not detect IRF7 activation in human NSCs upon poly(I:C) transfection. Therefore, IFN-α may not be as critical as IFN-β in antiviral response in human NSCs. In addition, IFN-stimulated antiviral genes, such as ISG56 and MxA, and the viral RNA sensors RIG-I, MDA5, and TLR3 can be expressed in NSCs after poly(I:C) transfection or IFN-β treatment. We further demonstrated that the cytoplasmic viral RNA sensors RIG-I and MDA5, as well as the endosomal PRR TLR3, play a role in the response to specific ligands for stimulating IFN-β transcription in NSCs. Furthermore, our results also show that infection of ZIKV or JEV to NSCs dramatically induces the expression of IFN-β. However, IFN-λ1, which is a type III IFN, was not significantly induced in ZIKV-infected human NSCs. This finding is consistent with previous publications describing that ZIKV does not induce type III IFN in human PBMCs and dendritic cells [45, 46] and that glioblastoma U-251 infected with ZIKV only induces 1–1.5-fold expression of IFN-λ1 [47]. The cytoplasmic PRR RIG-I plays a critical role in the induction of IFN-β. However, we found that MDA5 is also involved in the detection of JEV infection to induce IFN-β expression in NSCs. Collectively, this research demonstrates that human NSCs have the ability to activate IFN signals against neurotropic viral pathogens.

In the CNS, neural lineage cells, including neurons and astrocytes, have been demonstrated to produce IFN-β [48, 49], while oligodendrocytes produce limited type I IFN in response to poly(I:C) [50]. Our results further show that neural progenitor cells could also produce type I IFNs upon poly(I:C) stimulation or viral infection. Several cell types, such as pDCs and monocyte-derived DCs, have been demonstrated to produce IFN-λ1 during viral infection [51]. A previous study also showed that IFN-λ1 could be induced in human astrocytes in response to poly(I:C) via TLR3 [52]. Our results showed that stimulation with poly(I:C) not only triggers the transcription of IFN-β but also induces IFN-λ1 transcription in neural progenitor cells; hence, NSCs may be one of the cell types that produce IFN-λ1 in response to viral infections.

The cytoplasmic PRRs RIG-I and MDA5 are able to recognize specific pathogen RNA to induce IFN expression. However, a previous study showed that MDA5 is not expressed in human ESCs (hESCs) and that RIG-I is expressed but not responsive to its RNA ligand in hESCs [31]. In contrast, differentiated neural lineage cells, such as murine cortical neurons, possess functional RIG-I to mediate the production of proinflammatory cytokines in response to JEV infection [53]. Furthermore, differentiated human neuronal cells are also equipped with functional RIG-I and MDA5 to activate type I IFN expression upon poly(I:C) stimulation or Sendai virus infection [23]. Interestingly, a previous study suggested that knockdown of RIG-I expression decreased the proliferation of undifferentiated neural progenitor cells in JEV-infected animals caused by an increase in JEV replication and cell cycle inhibitors [54]. Nevertheless, our data indicate that human NSCs, like differentiated neural lineage cells, have functional RIG-I- and MDA5-mediated antiviral signaling pathways to respond to neurotropic virus infections.

In addition to cytosolic RLRs, endosomal TLR3, TLR7, and TLR8 are also capable of recognizing foreign nucleic acids to activate downstream innate immune responses. Few studies have investigated the expression and function of TLRs in neural progenitor cells. Previous studies revealed that the expression of TLR3 is decreased in the brain during embryo development. In addition, TLR3 signaling negatively regulates the proliferation of neural progenitor cells and hippocampal neurogenesis [34, 55]. Furthermore, the expression of TLR7 and TLR8 in the mouse brain is strongly regulated during development. The expression of TLR7 and TLR8 increases gradually from embryonic stages to postnatal days 4 and 21, respectively, and then subsequently declines [56, 57], and these TLRs play important roles in the regulation of neural differentiation [58, 59]. However, it was not clear whether these TLRs play roles in mediating innate immune responses in neural progenitor cells. We found that poly(A:U), the ligand of TLR3, was able to trigger IFN-β expression in those progenitor cells. Nevertheless, although TLR7 and TLR8 are expressed in NSCs, these TLRs lack the ability to induce type I IFN production in response to their specific ligands in the cells. These results imply that TLR7 and TLR8 may not be functional in activating the innate immune response in NSCs. Since previous studies have identified the role of TLR7 in the activation of the antiviral response in differentiated neural cells [23, 60], it is hypothesized that TLR7 plays a different role in neural progenitor cells.

Viral infections can be recognized by PRRs to trigger antiviral innate immune responses, such as the production of type I IFN. However, many viruses have developed strategies to subvert the host innate immune response. For example, NS1 and NS4B proteins of ZIKV are able to bind TBK1 and suppress the oligomerization of TBK1 and consequently limit the production of type I IFN, while NS2B-NS3 causes degradation of Jak1 protein and impairs the JAK-STAT pathway [61, 62]. Nonetheless, innate immune responses induced by a virus could be different among cell types. Our results show that infection with ZIKV dramatically induces IFN-β expression in NSCs and that induction or treatment with IFN-β is able to inhibit ZIKV replication in NSCs. In addition, the cytoplasmic pathogen sensor RIG-I mediates IFN-β transcription in ZIKV-infected NSCs. Since we have also found that treatment with IFN-β could induce downstream antiviral ISGs, such as ISG56 and MxA, in NSCs, this research clearly demonstrates that NSCs have intact RIG-I antiviral signaling pathways to interfere with viral propagation.

## Conclusion

Our data show that human neural progenitor cells can elicit type I and III IFNs and downstream antiviral ISGs and that the cytosolic RLRs RIG-I and MDA5, as well as the endosomal receptor TLR3, play an important role in the induction of IFNs. In addition, our results show that neural progenitor cells can be infected with neurotropic viruses, including ZIKV and JEV. This event may disrupt neuro-regeneration and result in pathogenesis in the CNS. Nevertheless, since we have also found that IFN-β can restrict the propagation of ZIKV and enhance the survival of NSCs, the administration of IFN may provide a treatment strategy to protect NSCs from infections by neurotropic viruses.

## Supplementary information

**Additional file 1: Figure S1.** Expression of RIG-I, MDA5, TLR3, ISG56 and MxA is upregulated in IFN-β-treated hNSCs. (A) RT-qPCR analysis was performed to detect the transcripts of RIG-I, MDA5, TLR3, ISG56 and MxA in human NSCs treated with 1 ng/ml IFN-β at the indicated time. The experiments were performed in triplicate, and the error bars represented the SD. (B) Immunoblot analysis was performed to examine the protein levels of RIG-I, MDA5 and TLR3 in 1 ng/ml IFN-β-treated hNSCs. β-actin was used as an internal control. (C) RT-qPCR analysis was performed to detect the mRNA levels of RIG-I, MDA5, TLR3, ISG56 and MxA in hNSCs treated with different doses of IFN-β for 2 h. The experiments were performed in triplicate, and the error bars represented the SD. (D) Immunoblot analysis was applied to detect protein levels of RIG-I, MDA5 and TLR3 in hNSCs treated with the indicated concentrations of IFN-β for 6 h.

**Additional file 2: Figure S2.** Expression of IFN-β is regulated by MDA5 and RIG-I in hNSCs . (A)Human NSCs and (B)HeLa cells were transfected with siRNA against RIG-I or MDA5 by Lipofectamine RNAiMAX 2000 and then transfected with 2 μg of total RNA extracted from EV-A71-infected Vero cells for 24 h. The expression of IFN-β mRNA was analyzed with RT-qPCR. Western blot was applied to confirm the knockdown efficiency. The experiments were performed in triplicate, and the error bars represented the SD. The Student’s *t* test was used for statistical analysis. *, *p* < 0.05, **, *p* < 0.01, ***, *p* < 0.001.

**Additional file 3: Figure S3.** RIG-I knockdown increases the replication of ZIKV in human NSCs. (A) Human NSCs were transfected with siRNA targeting RIG-I or MDA5 for 72 h, and then infected with ZIKV at an MOI of 1. Total RNA was collected to examine the mRNA expression of IFN-β using RT-qPCR. Western blot was performed to confirm the knockdown efficiency. (B) The expression of ZIKV vRNA was detected using RT-qPCR. (C) The viral growth curves were examined by performing plaque assay. The experiments were performed in triplicate, and the error bars represented the SD. The Student’s *t* test was used for statistical analysis. *, p<0.05, **, p < 0.01, ***, p < 0.001.

**Additional file 4: Figure S4.** RIG-I agonist 5’pppRNA inhibits ZIKV replication in hNSCs. (A-C) Human NSCs were transfected with 1 μg of 5’pppRNA for 6 h and then infected with ZIKV at an MOI of 1. Total RNA was harvested at 24 and 48 h post infection. The relative levels of IFN-β mRNA (A) and ZIKV virus RNA (vRNA) (B) were detected by using RT-qPCR. (C) Supernatants of the hNSCs were collected at 24 and 48 h post infection and virus titers were determined by plaque forming assay. The experiments were performed in triplicate, and the error bars represented the SD. The Student’s *t* test was used for statistical analysis. *, p<0.05, **, p < 0.01, ***, p < 0.001.

**Additional file 5.** **Table S1.** The primers used in the RT-qPCR assay.

## Data Availability

The data that support the findings of this study are available from the corresponding author upon reasonable request.

## Abbreviations

CNS: Central nervous system
NSC: Neural stem cell
MSC: Mesenchymal stem cells
PRR: Pattern recognition receptor
PAMP: Pathogen-associated molecular pattern
IFN: Interferon
TNF: Tumor necrosis factor
TLR: Toll-like receptor
RIG-I: Retinoic acid-inducible gene I
MDA5: Melanoma differentiation-associated protein 5
IRF3: Interferon regulatory factor 3
IRF7: Interferon regulatory factor 7
ISG: Interferon-stimulated gene
MxA: Myxovirus resistance gene A
5′ppp: 5′-Triphosphate
HMW: High molecular weight
PBMC: Peripheral blood mononuclear cells
pDC: Plasmacytoid dendritic cells
ZIKV: Zika virus
JEV: Japanese encephalitis virus
EV-A71: Enterovirus A 71
MOI: Multiplicity of infection
