# Supplementary figures and images for: Activation of type I interferon antiviral response in human neural stem cells
Source: Stem Cell Res Ther. 2019 Dec 16;10:387. doi: 10.1186/s13287-019-1521-5 (PMC6916114; doi:10.1186/s13287-019-1521-5)

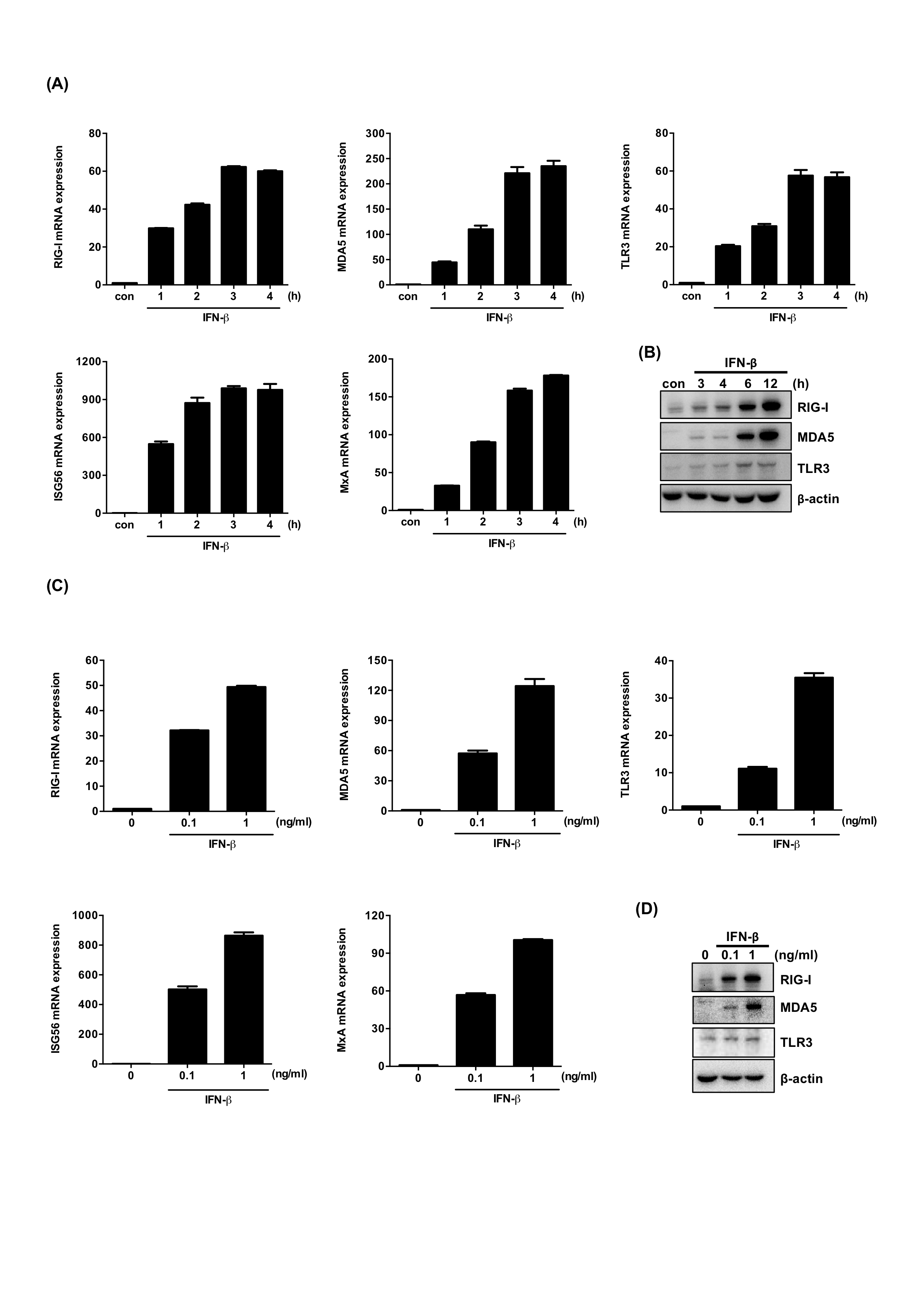

Supplement: Supplementary file 1 — Additional file 1: Figure S1. Expression of RIG-I, MDA5, TLR3, ISG56 and MxA is upregulated in IFN-β-treated hNSCs. (A) RT-qPCR analysis was performed to detect the transcripts of RIG-I, MDA5, TLR3, ISG56 and MxA in human NSCs treated with 1 ng/ml IFN-β at the indicated time. The experiments were performed in triplicate, and the error bars represented the SD. (B) Immunoblot analysis was performed to examine the protein levels of RIG-I, MDA5 and TLR3 in 1 ng/ml IFN-β-treated hNSCs. β-actin was used as an internal control. (C) RT-qPCR analysis was performed to detect the mRNA levels of RIG-I, MDA5, TLR3, ISG56 and MxA in hNSCs treated with different doses of IFN-β for 2 h. The experiments were performed in triplicate, and the error bars represented the SD. (D) Immunoblot analysis was applied to detect protein levels of RIG-I, MDA5 and TLR3 in hNSCs treated with the indicated concentrations of IFN-β for 6 h. [file 13287_2019_1521_MOESM1_ESM.tiff]

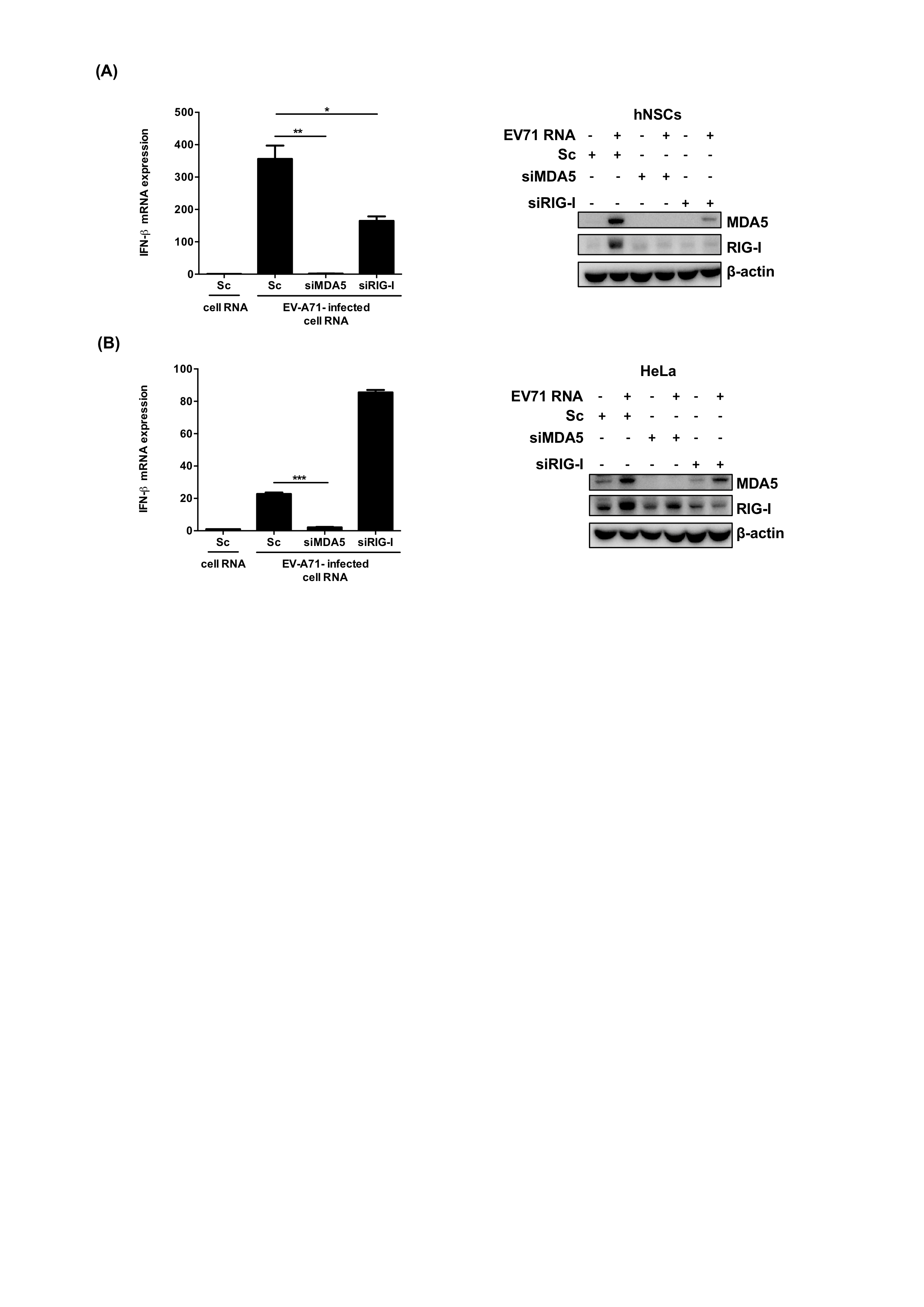

Supplement: Supplementary file 2 — Additional file 2: Figure S2. Expression of IFN-β is regulated by MDA5 and RIG-I in hNSCs . (A)Human NSCs and (B)HeLa cells were transfected with siRNA against RIG-I or MDA5 by Lipofectamine RNAiMAX 2000 and then transfected with 2 μg of total RNA extracted from EV-A71-infected Vero cells for 24 h. The expression of IFN-β mRNA was analyzed with RT-qPCR. Western blot was applied to confirm the knockdown efficiency. The experiments were performed in triplicate, and the error bars represented the SD. The Student’s t test was used for statistical analysis. *, p < 0.05, **, p < 0.01, ***, p < 0.001. [file 13287_2019_1521_MOESM2_ESM.tiff]

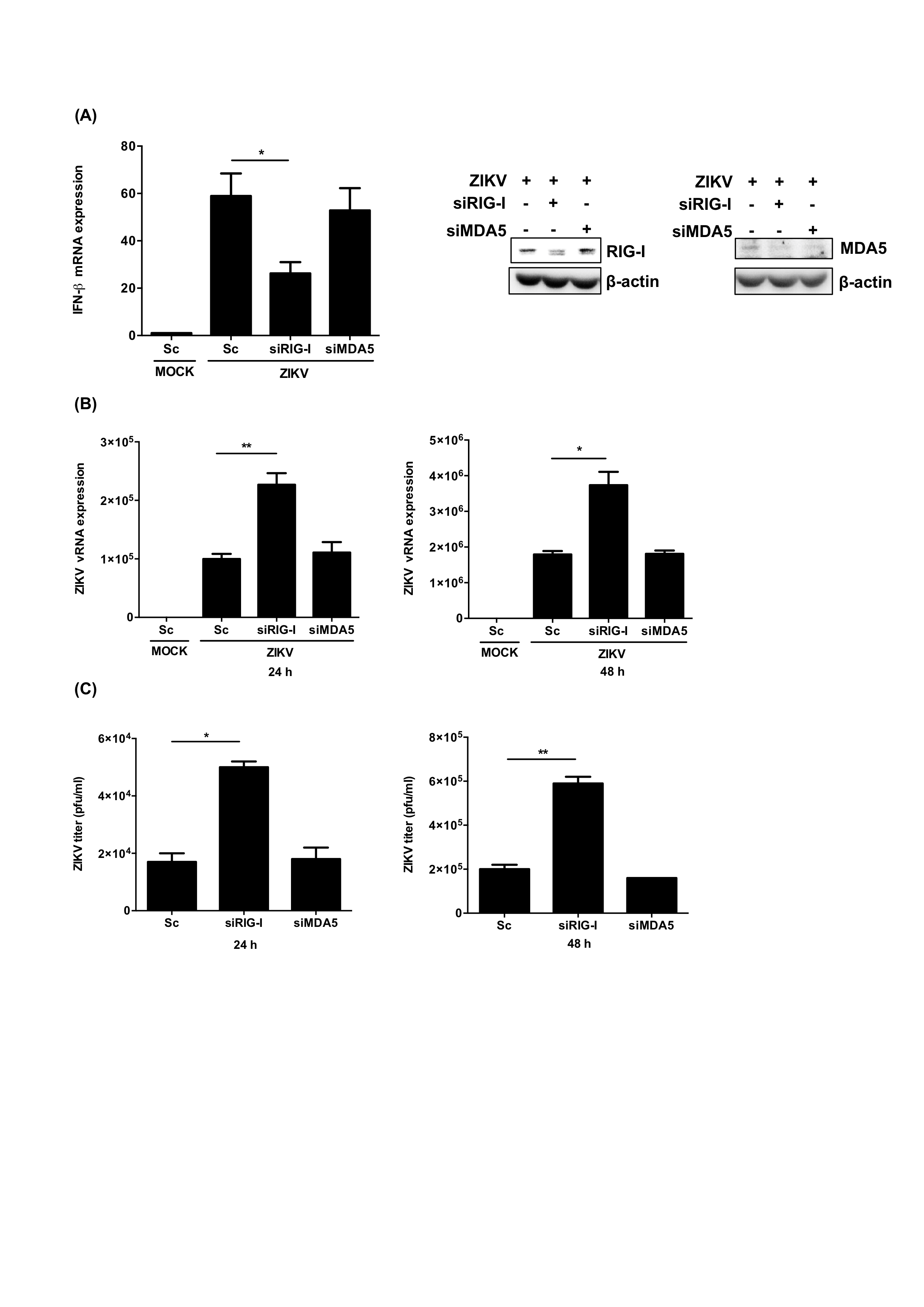

Supplement: Supplementary file 3 — Additional file 3: Figure S3. RIG-I knockdown increases the replication of ZIKV in human NSCs. (A) Human NSCs were transfected with siRNA targeting RIG-I or MDA5 for 72 h, and then infected with ZIKV at an MOI of 1. Total RNA was collected to examine the mRNA expression of IFN-β using RT-qPCR. Western blot was performed to confirm the knockdown efficiency. (B) The expression of ZIKV vRNA was detected using RT-qPCR. (C) The viral growth curves were examined by performing plaque assay. The experiments were performed in triplicate, and the error bars represented the SD. The Student’s t test was used for statistical analysis. *, p<0.05, **, p < 0.01, ***, p < 0.001. [file 13287_2019_1521_MOESM3_ESM.tiff]

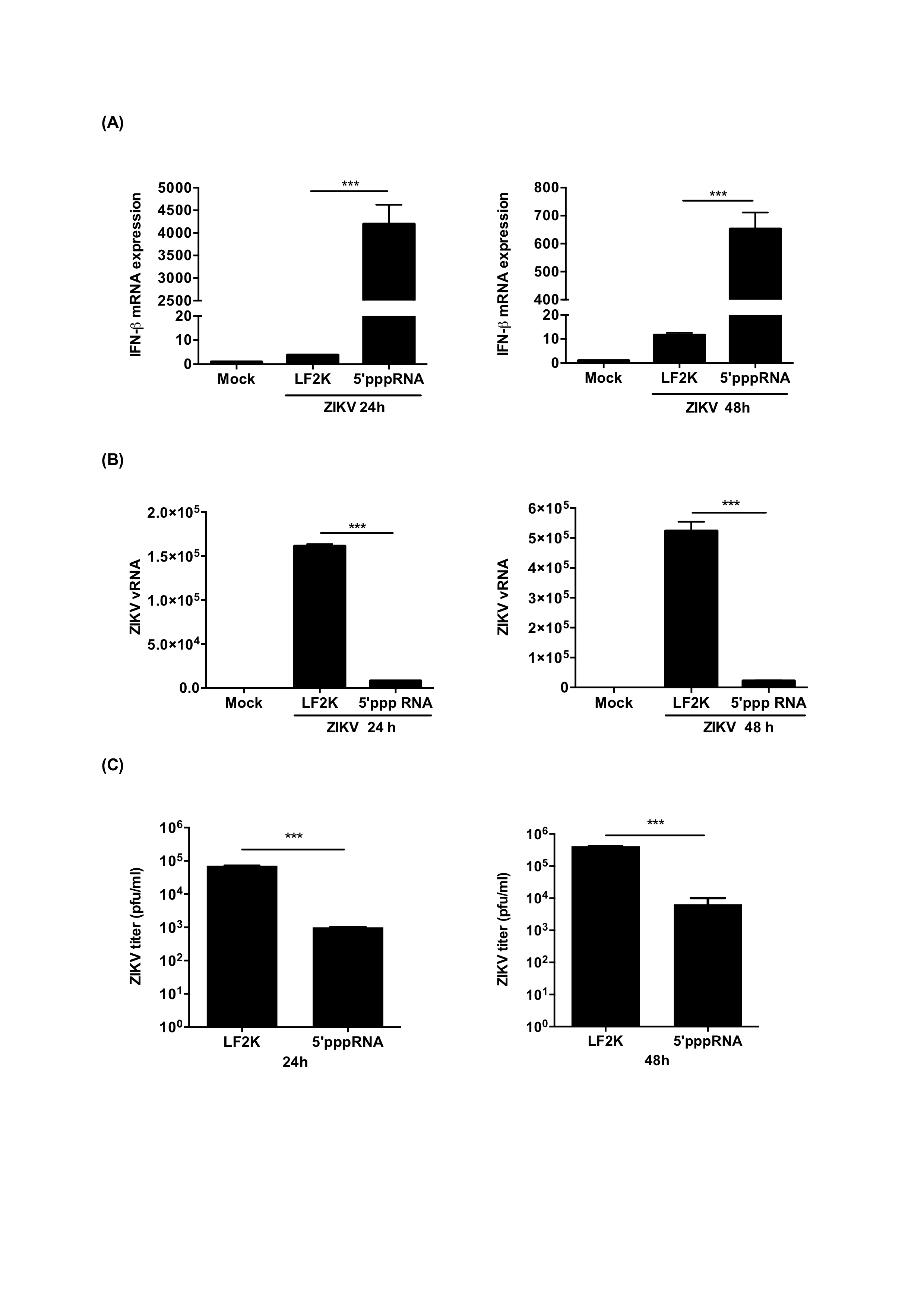

Supplement: Supplementary file 4 — Additional file 4: Figure S4. RIG-I agonist 5’pppRNA inhibits ZIKV replication in hNSCs. (A-C) Human NSCs were transfected with 1 μg of 5’pppRNA for 6 h and then infected with ZIKV at an MOI of 1. Total RNA was harvested at 24 and 48 h post infection. The relative levels of IFN-β mRNA (A) and ZIKV virus RNA (vRNA) (B) were detected by using RT-qPCR. (C) Supernatants of the hNSCs were collected at 24 and 48 h post infection and virus titers were determined by plaque forming assay. The experiments were performed in triplicate, and the error bars represented the SD. The Student’s t test was used for statistical analysis. *, p<0.05, **, p < 0.01, ***, p < 0.001. [file 13287_2019_1521_MOESM4_ESM.tiff]
