# Supplementary material for: Activation of type I interferon antiviral response in human neural stem cells
Source: Stem Cell Res Ther. 2019 Dec 16;10:387. doi: 10.1186/s13287-019-1521-5 (PMC6916114; doi:10.1186/s13287-019-1521-5)
Supplement: Supplementary file 5 — Additional file 5. Table S1. The primers used in the RT-qPCR assay. [file 13287_2019_1521_MOESM5_ESM.docx]

| Gene | Forward | Reverse |
| --- | --- | --- |
| IFN-α | GACTCCATCTTGGCTGTGA | TGATTTCTGCTCTGACAACCT |
| IFN-β | AGAAG GAGGA CGCCG CATTG | TCAGT TTCGG AGGTA ACCTG |
| IFN-λ1 | GTTCAAATCTCTGTCACCAC3 | TCACCTGGAGAAGCCTCAGG |
| TLR3 | AAG GGT GGC CCT TAA AAA TG | GTT TCC AGA GCC GTG CTA AG |
| MDA5 | AGGAGTCAAAGCCCACCATCTG | ATTGGTGACGAGACCATAACGGATA |
| RIG-I | GACCACATCCCAAGCCAAAG | TCATTTGGACATTTCTGCTG |
| MxA | TTCAGCACCTGATGGCCTATC | TGGATGATCAAAGGGATGTGG |
| ISG56 | TCTCAGAGGAGCCTGGCTAAG | CCACACTGTATTTGGTGTCTAGG |
| ZIKA NS2B | CTA GCG AAG TAC TCA CAG CTG T | GAC TTC CGC ATC TTT TTC CCA T |
| JEV | AGAACGGAAGATAACCATGACTAAA | CCGCGTTTCAGCATATTGAT |
| 18s rRNA | GTA ACC CGT TGA ACC CCA TT | CCA TCC AAT CGG TAG TAG CG |
